# Autoscopic Hallucinations in an African American Female Patient With Schizophrenia

**DOI:** 10.7759/cureus.13510

**Published:** 2021-02-23

**Authors:** Ganeya Gajaram, Tiffiney Lake, Dung Nguyen, Sukhjeet Sangha, Ayodeji Jolayemi

**Affiliations:** 1 Psychiatry, Interfaith Medical Center, Brooklyn, USA

**Keywords:** autoscopic hallucinations, autoscopy, visual hallucinations, schizophrenia, schizophrenia spectrum disorders, hallucinations

## Abstract

Autoscopic hallucinations are rare phenomena, with a handful of cases reported in patients with comorbidities and only one in a patient with schizophrenia. This case report discusses a 25-year-old African American female with schizophrenia and auditory hallucinations who presented with autoscopic hallucinations. The patient was interviewed on three separate occasions, her medical chart was consulted, a head computed tomography (CT) was performed, and her serum and urine laboratory values were monitored. Her head CT was normal, and her laboratory values were unremarkable. This case report contributes to the literature on autoscopic hallucinations in patients with schizophrenia.

## Introduction

Autoscopy is derived from the Greek words ‘autos,’ meaning “self,” and ‘skopeo,’ meaning “looking at” [1]. “Any man who believes he can see his own image appear in front of him when there is no mirror to reflect it is therefore subject to a visual hallucination of an autoscopic character,” postulated Lhermitte [2]. Autoscopic phenomena are dramatic visual own-body perceptions with experiences involving the replication of one’s own body in extrapersonal space generally when they are awake [1-7]. The patient maintains insight throughout the experience [1]. The descriptions of these hallucinations have been categorized into three distinct types: autoscopic hallucinations, heautoscopic hallucinations, and out-of-body experience [3-4,8]. During autoscopic hallucinations, a double of one’s own body is seen without any changes in bodily self-consciousness [9]. The observer’s perspective is body-centered and appears as a mirror reversal [3]. Lunn highlights that the most characteristic feature of this phenomenon is the patient’s visual experience: vivid, varied, and three-dimensional, where the patient is convinced of its reality and is usually scared or surprised at the vision [9]. Autoscopic hallucinations have been reported as polyopic, heterosexual, and even persecutory [3,5]. During out-of-body experiences, the second own body is visualized from an elevated perspective and location associated with disembodiment [4]. Heautoscopy describes seeing the self as a doppelganger [3], with strong self-identification with the double often associated with experiencing and perceiving the world from two places at the same time [3-4]. The double typically appears colorless, foggy, pale, through a veil, can behave autonomously, may mirror the person’s appearance, and can maintain sidedness [3]. Regardless of the type of autoscopy, these experiences may last for seconds, hours, and may even persist as a steady companion to the patient [3].

The etiology of autoscopic phenomena remains fodder for further research, however, numerous studies have reported their association with various conditions [1,3-4,6-9]. Autoscopic phenomena have been reported in numerous focal and generalized disorders of the central nervous system (CNS), during epileptic seizures, near-death experiences, meningitis, space-occupying lesions, brain tumors, migraine, delirium, posttraumatic brain lesions, multiple sclerosis, anxiety, sleep disturbances, either during or subsequent to substance use, alcohol withdrawal, medication side effect, infectious diseases like typhus, and altered psychological states [1-2,4,7-12]. Additionally, low glucose has been shown to induce autoscopic hallucinations in one case that was treated by correcting the glucose level [12]. Patients with schizophrenia have also been reported to experience autoscopy [1]. In fact, 16%-72% of patients with schizophrenia and schizoaffective disorder report a visual hallucination [11]. However, only one case has been reported to have autoscopic hallucinations [5]. Studies show that the global severity of illness is significantly higher in patients with schizophrenia and visual hallucinations as compared to those without [11]. Visual hallucinations in those with schizophrenia are generally vivid, involving family members, religious figures, and animals; the patient’s reactions can vary and include fear, pleasure, or indifference [11]. They are usually described as colorful, with normal-sized people and objects [11]. Alternatively, autoscopy has also been described in a patient with no psychiatric comorbidity [10].

We present the case of a patient who demonstrated autoscopic phenomena. The patient’s symptoms presented in the context of an acute exacerbation of schizophrenia. The significance of our findings are discussed in the context of literature regarding autoscopic hallucinations.

## Case presentation

A 25-year-old African American female with a past medical history of schizophrenia presented to the psychiatric emergency department (ED) due to an acute onset of auditory and visual hallucinations. She reported seeing herself outside of her body, stabbing herself, and felt helpless to stop it earlier that morning. She was seen smiling and laughing inappropriately and was unable to provide a clear timeline of her symptomatology. She denied any suicidal or homicidal ideation, with no intent or plan. She admitted to medication noncompliance of sertraline HCL and hydroxyzine of unknown dosages for an unknown period of time. She reported difficulty falling asleep and frequent night-time awakenings for the past several months. She denied thought insertion, thought broadcasting, ideas of reference, changes in appetite, energy, concentration, anhedonia, feelings of hopelessness, excessive guilt, worthlessness, increased irritability, excessive guilt, worthlessness, periods of increased energy with goal-directed activity, irritable mood, indiscretions, talkativeness, decreased need for sleep, racing thoughts, and anxiety. The patient also denied dysuria, frequency, and abnormal vaginal discharge. She reported no use of cigarettes, alcohol, and other recreational drugs. As per collateral from the patient's case manager, she reported the patient having interpersonal issues with her boyfriend, possibly a breakup, that could explain the worsening of her symptoms of auditory and visual hallucinations. The patient last received aripiprazole 400 mg intramuscular (IM) three weeks prior.

Upon evaluation, the patient appeared anxious but cooperative. She reported having auditory hallucinations for "a long time" but was unable to give details. She stated hearing male voices "telling good and bad things," endorsed visual hallucinations "seeing myself kicking and stabbing." She stated that the hallucinations had been bothering her lately. She also said that she had been using her coping skills, "grounding techniques", to overcome the perceptual disturbances that had not been effective. She reported feeling anxious, feeling shaky, could hear her heart pounding whenever she had to go out or be with people outside, feeling anxious and worried about doing presentations in school, and being scrutinized in public. She went on to state that her anxiety symptoms worsened when she was experiencing visual or auditory hallucinations. She recalled one previous suicide attempt where she lay down along the road in front of a car after being discharged from the hospital since she was not ready to be discharged but did not disclose when this occurred. The review of systems was positive for a history of anxiety and mild agoraphobia but negative for major depressive disorder, manic episodes, and post-traumatic stress disorder.

Upon further investigation into her psychiatric history, it was found that she had 29 documented visits in the last five years to two different hospitals for psychiatric complaints. Though the charts were not accessible, the summary of the visit showed that the patient was worked up for "schizoaffective bipolar type" and "unspecified psychosis." Her longest hospital stay was 485 days from July 13, 2015, to November 9, 2016, for "bipolar 1 depressed severe with psychotic features." No additional information could be obtained regarding her symptomatology or medication.

Medical history was unremarkable; she had no past surgical history, a head computed tomography (CT) was done in 2017 with a most recent head CT two weeks ago to rule out any brain abnormalities. No known medication or food allergies were noted. Current medications included aripiprazole, sertraline, and hydroxyzine pro re nata (PRN) of unknown dosages, which she had been noncompliant with.

The patient was unemployed, single, and received supplemental security income (SSI) benefits, which are managed by the group home. The SSI pays for her stay there as well as provides her with an allowance for food and other necessities. She graduated high school and is presently on her second attempt at college to earn a diploma. She identifies as a heterosexual woman and is in a relationship with a heterosexual man. She denies a family history of psychiatric illness, auditory hallucinations, and visual hallucinations.

The patient was anxious and jittery on physical exam but was otherwise unremarkable. She described her mood as “good,” which had improved after admission to the hospital. In regards to her perception, she was experiencing an auditory hallucination that she describes as hearing a man’s voice telling her she doesn’t matter. Visual hallucinations were also present: she sees herself spitting at other people as well as sees herself stabbing herself in the stomach. The patient otherwise had full insight into her illness. The mini-mental status exam (MMSE) was benign.

History of hallucinations

The auditory hallucinations began at around the age of 18-years-old. She recognized the voices as multiple women who sounded like family members who were telling her what to do; one, in particular, was to run away from home. After running away from home twice, she was hospitalized and treated for schizophrenia. When she was discharged this time, she was instructed to follow up with the primary care physician (PCP) as an outpatient and take the following medications: paliperidone palmitate, aripiprazole, and hydroxyzine hydrochloride, which she reported helped stop the voices when she was hospitalized. Information regarding dosages are unknown.

As per the patient, the voices returned after she was discharged from the hospital. The auditory hallucinations changed from voices of family members to screaming voices that were unknown to her. The auditory hallucinations later changed to a singular voice of a man, whose voice she did not recognize as well. This voice would tell her to harm herself and also spoke bad of others around her. She noted that the voices were loudest during the day and decreased at night when she is about to go to sleep. The patient is unable to recall if she had been compliant with her medications at that point in time.

A year after this man’s voice appeared, she started having visual hallucinations. Her first visual hallucination occurred while she was in a college class at the age of 19. She describes the visual hallucination as being vivid and in color and preceded by an urge to do something after which she would see herself performing the action. Examples of these hallucinations include seeing herself throw coffee at someone and one of the more common visual hallucinations was spitting on someone. The visions lasted less than a second, and she stated she was fully conscious and aware during the episode. She noted visual hallucinations were more frequent when she was anxious and occurred approximately three to five times daily. She did deny having any extraordinary abilities being carried out in the visual hallucinations such as flying or floating in midair. She also denied any mental, sexual, or physical childhood trauma and legal issues. She stated that she did well in school and is currently doing well in college though she dropped out two years ago because of her mental illness. She denied any past seizures or black-outs. The patient had good insight on her mental illness and drug treatment stating that “Abilify helps with the voices” and “Atarax and Zoloft help with decreasing the visions by reducing my anxiety.”

Hospitalization

Throughout this patient’s hospitalizations, her vitals remained stable and within reference ranges, with the exception of stable stage one hypertension. Her physical examination was consistently unremarkable throughout her hospitalization. The examination yielded no focal neurological deficits, with sensation grossly intact and motor graded at 5/5 in all the extremities. The patient's hematology and chemistry blood work panels were all within reference ranges.

Upon admission to the ED, the patient was started on aripiprazole 5 mg P.O. daily, Trazadone 50 mg per oral (PO) hora somni (HS) PRN for insomnia. Her scheduled medications include hydroxyzine HCL 25 mg PO twice a day (BID), lithium carbonate 600 mg PO HS, paliperidone palmitate 156 mg intramuscularly (IM) monthly, and propranolol HCL 10 mg PO BID. At that time, she was experiencing auditory hallucinations and was observed to be laughing inappropriately. On the first inpatient day, the patient continued to experience auditory hallucinations, visual hallucinations, appeared anxious, and was not oriented to person, time, or place. She was subsequently started on Haldol 5 mg PO daily for psychosis, Zoloft 50 mg PO for anxiety, Haldol 5 mg PRN q6h for agitation, and Ativan 2 mg PO q6h for agitation. Trazadone 50 mg PO HS PRN for insomnia was continued. On the second inpatient day, the patient was seen to be responding to internal stimuli, however, denied any auditory and visual hallucinations, paranoia, depression, and endorses feeling mild anxiety. Abilify 5 mg is discontinued and Haldol is titrated from 5 mg PO daily to 5 mg PO BID. She is continued on Zoloft 50 mg PO daily for her anxiety, Trazadone 50 mg PO PRN HS for insomnia, Haldol 5 mg PRN PO q6h for agitation, and Ativan 2 mg PO q6h PRN for agitation. On the third inpatient day, the patient is observed to be less internally preoccupied, has good insight, and a stable mood. Her medication regimen is continued. On the fourth inpatient day, there were no behavioral problems reported by the nursing staff, the patient reported that she was doing better, denied auditory and visual hallucinations, denied paranoia symptoms, and denied through insertion or ideas of reference. She reported that her sleep and appetite have been good in the hospital and denied feeling depressed or anxious. She denied any suicidal or homicidal thoughts, neither with intent nor plan. The patient also denied manic or depressive symptoms. She was continued on her medication regime. There was no note on this patient on the fifth inpatient day. On the sixth inpatient day, the patient reported no changes from the previous two days, and her medication regime was continued. She was compliant with her medications with no reported side effects. On the seventh inpatient day, the patient reported no changes from the previous day, was compliant with her medication, and expressed a plan of continuing her care with her outpatient psychiatrist after discharge. She was discharged on this day, as she was deemed psychiatrically stable.

**Figure 1:**
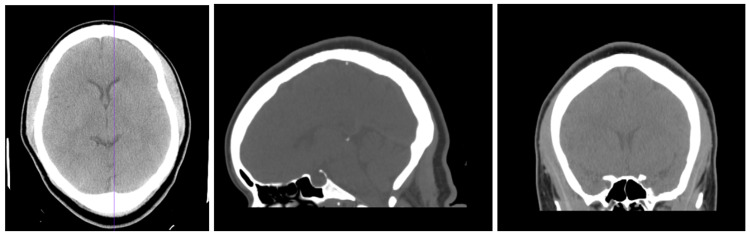
A noncontrast computed tomography of the patient’s head taken on November 9, 2020, one day prior to her discharge Image 1: Axial view of the patient's brain on computed tomography Image 2: Sagittal view of the patient's brain on computed tomography Image 3: Coronal view of the patient's brain on computed tomography

Differential diagnoses included substance-induced hallucinations and brain tumor-causing psychosis. Substance-induced hallucinations and psychosis were ruled out with a urine toxicology screen, which yielded negative results for opiates, methadone, propoxyphene, barbiturates, valproic acid, phencyclidine, amphetamines, benzodiazepines, cocaine, cannabinoids, and ethyl alcohol. A brain CT without contrast, as seen in Figure 1, was also obtained to rule out any tumor-inducing hallucinations. This scan included multiple contiguous axial images from the skull base to the vertex and both coronal and sagittal reconstructions were performed. The findings of this CT scan were determined to be normal. Additionally, questionnaires regarding adverse childhood experiences (Appendix A), trauma (Appendix B), and dissociation (Appendix C) were used to rule out a history of trauma and dissociation.

## Discussion

This case report presents a patient with a history of schizophrenia and auditory hallucinations who was experiencing autoscopic hallucinations. Her visual hallucinations were vivid and colorful where she was performing actions. Her hallucinations lasted a few seconds, happened in her immediate extracorporeal space, and were three-dimensional, all of which are consistent with previous reports on autoscopic hallucinations [3-4,8-9]. Her initial reaction was shock and surprise that other people around her cannot see her double, which had also been previously documented [9]. Furthermore, studies support the finding of autoscopic visual phenomena in patients with schizophrenia [1,11]. The patient did not fit the criteria for major depressive disorder, generalized anxiety disorder, and the Dissociative Experiences Scale, thereby ruling out depression, anxiety, and dissociation. Her Adverse Childhood Experiences Scale (ACES) score was zero, which indicates there were no signs of childhood trauma. She also did not meet the criteria for posttraumatic stress disorder and the Brief Trauma Questionnaire proved unremarkable. Table 1 lists the classification criteria for heautoscopy, out-of-body experience, and autoscopic hallucinations, as well as lesion location.

**Table 1:** Classification criteria for heautoscopy, out-of-body experience, and autoscopic hallucinations, as well as lesion location suggested by previous case reports and small case series is shown Source: [4]

	Autoscopic Hallucination	Out-of-Body Experience	Heautoscopy
Self-location	Centered at physical body, stable	Centered at illusory body, stable	Centered at physical and/or illusory body, unstable
Self-identification	With physical body	With illusory body	With physical and/or illusory body
First-person perspective	Centered at physical body, stable	Centered at illusory body, stable	Centered at physical and/or illusory body, unstable
Second own body (autoscopic body)	2D image of own body, often of the face and upper trunk	3D image of whole own body	3D image of whole own body
Vividness/realism	Low	High	High
Lesion location	Bilateral, occipital, temporal	Right temporal, Parietal	Left, temporal, Parietal

It was noted, however, that she had experienced recent emotional stress due to issues with her partner and that this may have triggered the acute exacerbation of her hallucinations, coupled with her medication noncompliance. The literature has described patients both with and without additional psychiatric comorbidities. This case report supports the scant literature on the latter.

Though it has been reported that patients with neurological and psychiatric diseases like epileptic seizures, near-death experiences, meningitis, space-occupying lesions, brain tumors, migraines, delirium, posttraumatic brain lesions, multiple sclerosis, anxiety, sleep disturbances, substance use, alcohol withdrawal, medication side effect, infectious diseases like typhus, and altered psychological states have experienced autoscopic phenomena, the patient denies any history of the aforementioned conditions [1-2,4,7-12]. Furthermore, several cortical areas have been implicated in autoscopy either in the capacity of damage or hypofunction: the temporoparietal junction, the vestibular system, right occipital cortex, nondominant gyrus angularis, and extrastriate cortex [3-4,12]. CT of the head performed on the patient revealed no structural abnormalities in these regions. One report described low glucose-inducing autoscopic hallucinations in one case that was treated by correcting the glucose level [12]. This patient’s blood glucose was 116 mg/dL, and her glycated hemoglobin (HbA1C) was 5.9% on November 3, 2020. The only other case of autoscopic hallucination in a patient with Schizophrenia presented similarly to our patient in many regards: auditory hallucinations preceding for a few years before the onset of visual hallucinations of a persecutory type, visualize autoscopic hallucination a few feet away from them, seeing visions of bad things happening and feeling guilty that they are unable to intervene, improvement of autoscopic hallucinations on antipsychotic medications - Risperidone up to 6 mg/day for the other case and Abilify Maintena 400 mg (aripiprazole), Zoloft 50 mg (sertraline) q12, Atarax (hydroxyzine) PRN in our patient, and similar age - our patient, 25, and the other case, 27 [5]. Marked differences between the two include: occurring spontaneously without any identifiable trigger or pattern in the other case where our patient develops them during periods of stress.

There has only been one case of autoscopic hallucinations in a patient with schizophrenia that has been reported [5] and several cases of autoscopy with comorbidities [7,10]. This case report contributes to the scarce literature on autoscopic hallucinations and is the first of its kind to report on an African American female patient experiencing autoscopic hallucinations preceded by auditory hallucinations with a history of schizophrenia.

Though the pathophysiology of autoscopy is not cemented, several theories have been postulated in the literature, some with support from the data [3,11]. One theory is the failure to integrate multisensory signals at the temporoparietal junction leading to a collapse of the spatial unity between the self and the body [3]. In fact, cerebral lesions at the temporoparietal junction or in adjacent regions have been identified in a sample with autoscopic phenomena [8]. Another postulates that heautoscopy is based on abnormal integration of multisensory signals in personal space as well as extrapersonal space [4]. Though the contribution of visual and somatosensory input of self-location is supported by clinical and experimental data, much remains a mystery about the role of the vestibular system [3]. Studies have found that heautoscopy was associated with lesions to the left posterior insula, autoscopic hallucinations with damage to the right occipital cortex, and out-of-body experiences with the temporoparietal cortex [4]. There is some data suggesting that autoscopic hallucinations are a result of visuo-somatosensory deficit associated with the extrastriate cortex and not associated with major deficits in bodily self-consciousness despite the visual hallucination of the own body [4]. Additionally, there is much clinical evidence on the importance of emotional and interoceptive signal processing in the posterior insula in relation to bodily self-consciousness.

Other theories postulate the involvement of the primary visual cortex, namely, Brodmann’s area 17, which may be irritated due to seizure activity, the reticular activating system, and lesions in the brainstem [11].

Our patient showed no clear evidence of pathology on CT imaging of the brain when contrasted to the findings in the literature. This, however, does not rule out the possibility of brain pathology that may only be apparent on positron emission tomography (PET) scan or functional MRI. The need to perform these images on our patient may be one limitation, as it could provide a much clearer picture of the pathophysiology of this unusual hallucination in our patient. This case adds to the reports of the phenomena of autoscopic hallucinations and there is a need to identify the potential pathogenesis of autoscopic hallucinations, both to identify which patients may present with these symptoms and for therapeutic purposes.

We explored the different presentations of autoscopic hallucinations in cases reported in the literature. We also explored the various locations of brain lesions, metabolic, and organic causes of autoscopy. We conducted a literature review using PubMed, Google Scholar, and OMNI Queens Library Online database using the keywords “autoscopy,” “visual hallucinations,” “autoscopic hallucinations,” and “hallucinations of self” to find articles in these databases. Language and timeframe were restricted due to the paucity of articles on this topic. Peer-reviewed case reports discussing psychiatric symptoms were reviewed by the five authors. Articles included in the report ranged in date from 1964-2019. Any disagreements regarding the eligibility of an article were resolved by discussion among the authors. Limitations to our report include missing information regarding the patient's stay on day five, incomplete notes in her electronic medical record, and lack of access to her previous psychiatric hospitalization records.

## Conclusions

Autoscopic hallucinations are scarcely seen in clinical practice and even less documented in the literature. This case report highlights this rare phenomenon of visualizing the self outside of the body with no fluctuation in consciousness in the absence of substance-induced psychosis, metabolic derangements, or structural abnormalities within the brain. This paper contributes to the literature on specific types of auditory and visual hallucinations that can be experienced by a patient with schizophrenia. Further studies are needed to examine the neurobiological pathophysiology of autoscopic hallucinations, examine the psychosocial determinants that may contribute to its manifestation, and strategies to alleviate them.

## Appendices

Appendix A 

**Table 2:** Adverse Childhood Experiences Score (ACES) Questionnaire that was conducted during an interview with the patient

Question Prior to 18th Birthday:	no	yes	#
Did a parent or other adult in the household often or very often… Swear at you, insult you, put you down, or humiliate you? or Act in a way that made you afraid that you might be physically hurt?	✓		0
Did a parent or other adult in the household often or very often… Push, grab, slap, or throw something at you? or ever hit you so hard that you had marks or were injured?	✓		0
Did an adult or person at least 5 years older than you ever… Touch or fondle you or have you touch their body in a sexual way? or Attempt or actually have oral, anal, or vaginal intercourse with you?	✓		0
Did you often or very often feel that … No one in your family loved you or thought you were important or special? or Your family didn’t look out for each other, feel close to each other, or support each other?	✓		0
Did you often or very often feel that … You didn’t have enough to eat, had to wear dirty clothes, and had no one to protect you? or Your parents were too drunk or high to take care of you or take you to the doctor if you needed it?	✓		0
Were your parents ever separated or divorced?	✓		0
Was your mother or stepmother: Often or very often pushed, grabbed, slapped, or had something thrown at her? or Sometimes, often, or very often kicked, bitten, hit with a fist, or hit with something hard? or Ever repeatedly hit over at least a few minutes or threatened with a gun or knife?	✓		0
Did you live with anyone who was a problem drinker or alcoholic, or who used street drugs?	✓		0
Was a household member depressed or mentally ill, or did a household member attempt suicide?	✓		0
Did a household member go to prison?	✓		0

Appendix B 

**Table 3:** Brief Trauma Questionnaire that was conducted with the patient during an interview

Event	Has this ever happened to you?	If the event happened to you, did you think your life was in danger or you might be seriously injured?	If the event happened were you seriously injured?
Have you ever served in a war zone, or have you ever served in a non-combat job that exposed you to war-related casualties (for example, as a medic or on graves registration duty?)	No	N/A	N/A
Have you ever been in a serious car accident, or a serious accident at work or somewhere else?	No	N/A	N/A
Have you ever been in a major natural or technological disaster, such as a fire, tornado, hurricane, flood, earthquake, or chemical spill?	No	N/A	N/A
Have you ever had a life-threatening illness such as cancer, a heart attack, leukemia, AIDS, multiple sclerosis, etc.?	No	N/A	
Before age 18, were you ever physically punished or beaten by a parent, caretaker, or teacher so that: you were very frightened; or you thought you would be injured; or you received bruises, cuts, welts, lumps, or other injuries?	No	N/A	N/A
Not including any punishments or beatings you already reported in Question 5, have you ever been attacked, beaten, or mugged by anyone, including friends, family members, or strangers?	No	N/A	N/A
Has anyone ever made or pressured you into having some type of unwanted sexual contact? Note: By sexual contact, we mean any contact between someone else and your private parts or between you and some else’s private parts	No	N/A	N/A
Have you ever been in any other situation in which you were seriously injured, or have you ever been in any other situation in which you feared you might be seriously injured or killed?	No		N/A
Has a close family member or friend died violently, for example, in a serious car crash, mugging, or attack?	No		N/A
Have you ever witnessed a situation in which someone was seriously injured or killed, or have you ever witnessed a situation in which you feared someone would be seriously injured or killed? Note: Do not answer “yes” for any event you already reported in Questions 1-9	No		

Appendix C 

**Table 4:** Dissociative Experiences Scale conducted with the patient during an interview

Question	Response	Total
Some people have the experience of driving or riding in a car or bus or subway and suddenly realizing that they don’t remember what has happened during all or part of the trip. Select the number to show what percentage of the time this happens to you. (0% Never, 100% Always)	0	0
Some people find that sometimes they are listening to someone talk and they suddenly realize that they did not hear part or all of what was said. Select the number to show what percentage of the time this happens to you. (0% Never, 100% Always)	0	0
Some people have the experience of finding themselves in a place and have no idea how they got there. Select the number to show what percentage of the time this happens to you. (0% Never, 100% Always)	0	0
Some people have the experience of finding themselves dressed in clothes that they don’t remember putting on. Select the number to show what percentage of the time this happens to you. (0% Never, 100% Always)	0	0
Some people have the experience of finding new things among their belongings that they do not remember buying. Select the number to show what percentage of the time this happens to you. (0% Never, 100% Always)	0	0
Some people sometimes find that they are approached by people that they do not know, who call them by another name or insist that they have met them before. Select the number to show what percentage of the time this happens to you. (0% Never, 100% Always)	0	0
Some people sometimes have the experience of feeling as though they are standing next to themselves or watching themselves do something and they actually see themselves as if they were looking at another person. Select the number to show what percentage of the time this happens to you. (0% Never, 100% Always)	0	0
Some people are told that they sometimes do not recognize friends or family members. Select the number to show what percentage of the time this happens to you. (0% Never, 100% Always)	0	0
Some people find that they have no memory for some important events in their lives (for example, a wedding or graduation). Select the number to show what percentage of the time this happens to you. (0% Never, 100% Always)	0	0
Some people have the experience of being accused of lying when they do not think that they have lied. Select the number to show what percentage of the time this happens to you. (0% Never, 100% Always)	0	0
Some people have the experience of looking in a mirror and not recognizing themselves. Select the number to show what percentage of the time this happens to you. (0% Never, 100% Always)	0	0
Some people have the experience of feeling that other people, objects, and the world around them are not real. Select the number to show what percentage of the time this happens to you. (0% Never, 100% Always)	0	0
Some people have the experience of feeling that their body does not seem to belong to them. Select the number to show what percentage of the time this happens to you. (0% Never, 100% Always)	0	0
Some people have the experience of sometimes remembering a past event so vividly that they feel as if they were reliving that event. Select the number to show what percentage of the time this happens to you. (0% Never, 100% Always)	0	0
Some people have the experience of not being sure whether things that they remember happening really did happen or whether they just dreamed them. Select the number to show what percentage of the time this happens to you. (0% Never, 100% Always)	0	0
Some people have the experience of being in a familiar place but finding it strange and unfamiliar. Select the number to show what percentage of the time this happens to you. (0% Never, 100% Always)	0	0
Some people find that when they are watching television or a movie they become so absorbed in the story that they are unaware of other events happening around them. Select the number to show what percentage of the time this happens to you. (0% Never, 100% Always)	0	0
Some people find that they become so involved in a fantasy or daydream that it feels as though it were really happening to them. Select the number to show what percentage of the time this happens to you. (0% Never, 100% Always)	0	0
Some people find that they sometimes are able to ignore pain. Select the number to show what percentage of the time this happens to you. (0% Never, 100% Always)	0	0
Some people find that they sometimes sit staring off into space, thinking of nothing, and are not aware of the passage of time. Select the number to show what percentage of the time this happens to you. (0% Never, 100% Always)	0	0
Some people sometimes find that when they are alone they talk out loud to themselves. Select the number to show what percentage of the time this happens to you. (0% Never, 100% Always)	0	0
Some people find that in one situation they may act so differently compared with another situation that they feel almost as if they were two different people. Select the number to show what percentage of the time this happens to you. (0% Never, 100% Always)	0	0
Some people sometimes find that in certain situations they are able to do things with amazing ease and spontaneity that would usually be difficult for them (for example, sports, work, social situations, etc.). Select the number to show what percentage of the time this happens to you. (0% Never, 100% Always)	0	0
Some people sometimes find that they cannot remember whether they have done something or have just thought about doing that thing (for example, not knowing whether they have just mailed a letter or have just thought about mailing it). Select the number to show what percentage of the time this happens to you. (0% Never, 100% Always)	0	0
Some people find evidence that they have done things that they do not remember doing. Select the number to show what percentage of the time this happens to you. (0% Never, 100% Always)	0	0
Some people sometimes find writings, drawings, or notes among their belongings that they must have done but cannot remember doing. Select the number to show what percentage of the time this happens to you. (0% Never, 100% Always)	0	0
Some people sometimes find that they hear voices inside their head that tell them to do things or comment on things that they are doing. Select the number to show what percentage of the time this happens to you. (0% Never, 100% Always)	100	100
Some people sometimes feel as if they are looking at the world through a fog, so that people and objects appear far away or unclear. Select the number to show what percentage of the time this happens to you. (0% Never, 100% Always)	0	0
